# Clinical, Biochemical, and Molecular Characteristics of Filipino Patients with Tyrosinemia Type 1

**DOI:** 10.3390/ijns10030059

**Published:** 2024-08-31

**Authors:** Barbra Charina V. Cavan, Leniza G. de Castro-Hamoy, Conchita G. Abarquez, Ebner Bon G. Maceda, Maria Melanie Liberty B. Alcausin

**Affiliations:** 1 Center for Human Genetics Services, Institute of Human Genetics, National Institutes of Health, University of the Philippines Manila, Pedro Gil St., Ermita, Manila 1000, Philippines; bvcavan@up.edu.ph (B.C.V.C.); lgdecastrohamoy@up.edu.ph (L.G.d.C.-H.); egmaceda@up.edu.ph (E.B.G.M.); 2 Department of Pediatrics, Philippine General Hospital, University of the Philippines Manila, Pedro Gil St., Ermita, Manila 1000, Philippines; 3 Newborn Screening Center Mindanao, Southern Philippines Medical Center, J.P. Laurel Ave, Bajada, Davao City 8000, Philippines; cgabarquez@nscmindanao.ph

**Keywords:** tyrosinemia type I, hepatorenal tyrosinemia, expanded newborn screening

## Abstract

Hereditary tyrosinemia type I (HT1), or hepatorenal tyrosinemia, is an amino acid disorder which may cause hepatic failure as well as renal and neurologic comorbidities. Early detection of this disorder is possible with newborn screening (NBS). The objective of this study is to describe the clinical, biochemical, and molecular characteristics of Filipino patients diagnosed with HT1 through the expansion of the Philippine NBS program in 2014. There were a total of 16 patients with confirmed HT1 from then until September 2022. Clinical and biochemical data during confirmation and initial evaluation, as well as molecular data, were obtained from the patients’ medical records. The cohort included children between the ages of 18 and 54 months at the time of data collection. The mean age at treatment initiation was 26.8 days. The mean succinylacetone level from dried blood spot sampling using tandem mass spectrometry (MS) was 11.1 µmol/L. Biochemical confirmatory tests via plasma amino acid analysis showed mean levels of tyrosine, phenylalanine, and methionine of 506.1 µmol/L, 111.5 µmol/L, and 125.4 µmol/L, respectively. Upon urine organic acid (UOA) analysis, succinylacetone was detected in all except for one patient, who was managed prior to UOA analysis. The most common clinical characteristics were abnormal clotting times (62.5%), elevated alpha fetoprotein (37.5%), anemia (31.3%), and metabolic acidosis (31.3%). The most common genotype was homozygous c.122T>C p.Leu41Pro in 64.3% of patients. The allelic frequency of this pathogenic variant is 71.4%. The inclusion of HT1 in the Philippine NBS program allowed early diagnosis and management of HT1 patients.

## 1. Introduction

**Figure 1:**
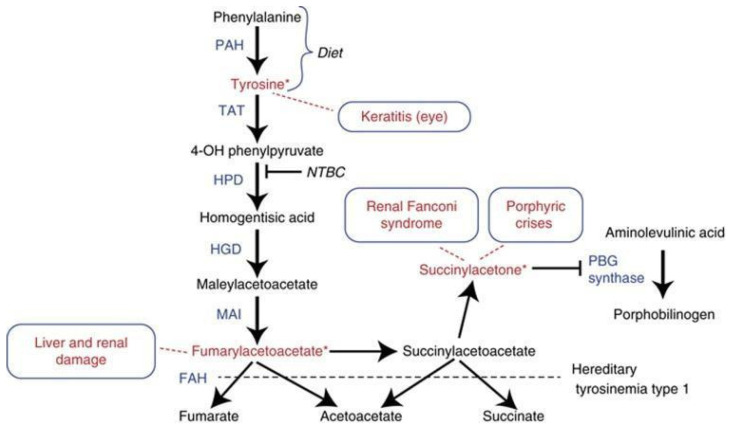
The abnormalities in tyrosinemia type 1 depicted in the pathway of tyrosine metabolism [2].

Hereditary tyrosinemia type 1 (HT1), also known as hepatorenal tyrosinemia, is a rare autosomal recessive inborn error of metabolism. It is caused by a defect in the final enzyme of the degradation pathway of tyrosine, named fumarylacetoacetate hydroxylase (FAH) (Figure 1). As a result of the metabolic block, toxic metabolites are formed, including succinylacetone (SA), maleylacetoacetate, and fumarylacetoacetate. These metabolites are responsible for the severe disruption of the intracellular metabolism of the liver and kidney [1].

Symptoms typically present within the first two years of life and include acute liver failure, renal dysfunction, and, in a few cases, neurologic crisis [2]. Untreated patients have a high risk of developing hepatocellular carcinoma. The estimated worldwide incidence of HT1 is about 1 in 100,000, being more common in the Saguenay–Lac-St. Jean area of Quebec with an incidence of 1 in 1846 [3].

The FAH gene responsible for HT1 is located in chromosome 15q25.1, spanning 35 kb, and has 14 exons, encoding a 419 amino acid protein [4]. The deficiency of FAH gives rise to elevated plasma concentrations of amino acids phenylalanine, tyrosine, and methionine, as well as excretion of unusual tyrosine metabolites like SA, which is a highly sensitive and specific marker for HT1, along with elevated urinary concentrations of tyrosine metabolites (4-hydroxyphenylpyruvate, 4-hydroxyphenyllactate, and 4-hydroxyphenylacetate) [5]. Succinylacetone remains to be the best marker for HT1, as it is pathognomonic for HT1 and tyrosine is not always elevated in all HT1 cases [6]. Recommendations for the confirmation of the condition after a positive newborn screening are blood or urine SA, plasma amino acids, liver function tests, and alpha fetoprotein [2]. Genetic testing of the FAH gene for biallelic pathogenic variants can also confirm the diagnosis in more than 95% of cases [7].

Medical management of HT1, including dietary management and initiation of nitisinone, should commence as soon as the diagnosis is confirmed. The drug of choice is nitisinone, also called 2-(2-nitro-4-trifluoromethyl benzoyl)-1,3-cyclohexadiene or NTBC, which acts by blocking the parahydroxyphenylpyruvic acid dioxygenase (*p*-HPPD) enzyme in the second step of the tyrosine degradation pathway. This prevents the accumulation of fumarylacetoacetate and its conversion to SA. Since the blood concentration of tyrosine remains elevated, dietary management with controlled intake of phenylalanine and tyrosine should also be initiated immediately after diagnosis. Before nitisinone became available, the only definitive therapy for tyrosinemia type I was liver transplantation. Current recommendations, however, are for liver transplantation to be reserved for the children who have severe liver failure at presentation and fail to respond to nitisinone, or have evidence of malignancy in their hepatic tissue [5].

The Philippine newborn screening program is a government-run program by the Department of Health. There are seven strategically located newborn screening centers all over the country that receive and run dried blood spot samples five days a week. Each center has a follow-up medical team that informs the birth facilities of positive screens and conducts active recall. If necessary, in symptomatic cases, each medical team is trained to start acute treatment prior to confirmatory tests.

In the Philippines, the original six disorder panel (glucose 6 phosphate dehydrogenase deficiency, congenital hypothyroidism, congenital adrenal hyperplasia, galactosemia, maple syrup urine disease, and phenylketonuria) screened via NBS was expanded in December 2014 to include other metabolic conditions via tandem mass spectrometry (MS/MS) [6]. The kit used for the HT1 screening in the Philippines is the Neobase Non-derivatized MS/MS Kit, using succinylacetone as the primary marker. The kit is supplied with a full complement of internal standards for the measurement of succinylacetone and 11 amino acids (Ala, Arg, Cit, Gly, Leu/Ile/Pro-OH, Met, Orn, Phe, Pro, Tyr, Val). For SA, the internal standard is ^13^C_5_-MPP* and the control is SA. In 2019, full insurance coverage for the expanded panel assured that all newborns who undergo screening are tested for more than 28 disorders, including HT1.

This study aimed to describe the clinical, biochemical, and molecular characteristics of Filipino patients diagnosed with HT1 through the Philippine NBS program. Specifically, the objectives were to determine the clinical and biochemical characteristics of patients diagnosed with HT1 around the time of initial evaluation; describe the pathogenic variants seen in this cohort of patients; and determine the period prevalence of HT1 in the Philippines from December 2014 to September 2022.

## 2. Materials and Methods

This study utilized a retrospective, descriptive study design. Approval from the institutional ethics review board was sought prior to data collection. The medical records of patients who underwent NBS from December 2014 to September 2022 and were biochemically and molecularly confirmed as HT1 cases were reviewed. The data were obtained from the Newborn Screening Reference Center (NSRC), which keeps the national data from all seven newborn screening centers, and the Institute of Human Genetics (IHG), which houses the confirmatory laboratories. Both units are under the National Institutes of Health, University of the Philippines Manila (UP-NIH).

A total of 16 confirmed cases of HT1 screened by NBS using tandem mass spectrometry and confirmed via biochemical studies and/or molecular studies were reviewed in this study. The study did not include patients diagnosed with tyrosinemia other than HT1 or screen-positive babies without confirmatory tests. Data on the patients’ anthropometrics, date of birth, age when NBS was performed, presence or absence of symptoms, results of NBS, plasma amino acid profile, urine organic acid profile, and molecular test results were obtained from the medical records using a standardized data-collection form. All biochemical tests were completed at the Biochemical Genetics Laboratory at IHG, UP-NIH. The plasma amino acid analysis utilized high-performance liquid chromatography (HPLC), while urine organic acid analysis was conducted through gas chromatography–mass spectrometry (GCMS). Genetic test results were performed using next generation sequencing (NGS), either through Invitae or Fulgent Laboratories (in the USA).

## 3. Results

There were a total of 16 patients diagnosed with HT1 in the Philippines detected via NBS from December 2014 to September 2022. Of these, 14 patients are alive and 2 had an untimely demise due to infectious causes.

### 3.1. General Characteristics and Biochemical Profile of Filipino Patients with Tyrosinemia Type 1

The cohort of 16 patients were between the ages of 18 and 54 months at the time of data collection. There were nine males and seven females. The mean age at treatment initiation with NTBC was 26.8 (±19.4) days. The mean SA level via NBS was 11.1 (±4.1) µmol/L, where the lab cut-off was <5 µmol/L. The mean plasma tyrosine level on the amino acid profile was 506.1 µmol/L (reference range: 0–30 days: 55–147; 31 days–23 mos: 22–108). Mean phenylalanine level (reference range: 0–30 days: 38–137; 31 days–23 mos: 31–75) and mean methionine level (reference range: 0–30 days: 10–60; 31 days–23 mos: 9–42) were 111.5 µmol/L and 125.4 µmol/L, respectively. Upon semi-quantitative urine organic acid (UOA) analysis, SA was present except for patient 4, who was confirmed molecularly and was already on dietary treatment and NTBC when the urine sample for UOA analysis was collected (Table 1).

### 3.2. Clinical Characteristics of Filipino Patients with Tyrosinemia Type 1

The most common clinical characteristics reported in the charts were abnormal clotting times based on prothrombin time (PT) and partial thromboplastin time (PTT), elevated alpha fetoprotein, anemia, and acidosis at 62.5%, 37.5%, 31.3%, and 31.3%, respectively (Table 2). These clinical characteristics were noted around the time of initiation of treatment. Medical records of the live patients who were all on dietary and medical treatment showed absence of jaundice, ascites, seizures, or symptoms of liver or kidney disease.

### 3.3. Genotypic and Allelic Frequencies of FAH Pathogenic Variants in Filipino Patients with Tyrosinemia Type 1 in the Study

#### 3.3.1. Genotypic Frequencies of FAH Pathogenic Variants in Filipino Patients with Tyrosinemia Type 1

Fourteen of the patients with HT1 underwent molecular confirmation. Twelve were homozygotes and two were compound heterozygotes. The most common genotype was the homozygous c.122T>C (p.Leu41Pro) pathogenic variant in 9 out of 14 patients (64.3%). In addition, both compound heterozygotes had the c.122T>C p.Leu41Pro pathogenic variant in the alleles (Table 3).

#### 3.3.2. Allelic Frequencies of FAH Pathogenic Variants in Filipino Patients with Tyrosinemia Type 1

There were five pathogenic variants noted in this cohort. The most common FAH pathogenic variants were c.122T>C (p.Leu41Pro) and c.535_536del (p.Gln179Aspfs*4), at 71.4% and 10.7%, respectively (Table 4).

## 4. Discussion

This retrospective study is the first to describe the cohort of Filipino patients with HT1. These patients were diagnosed through expanded NBS. One case was previously reported [8]. There were no patients with HT1 in the Philippines detected outside of the NBS program. This study describes 16 patients from 16 unrelated families. Two patients had consanguineous parents. The first patient with HT1 was diagnosed in 2019; since then, an average of four babies were diagnosed with HT1 in the Philippines annually.

The initiation of treatment, through both dietary management and nitisinone, was early in this cohort because all were detected via newborn screening. Hence, the clinical picture observed is very different from the classic description of the natural history of HT1. It was noted that children younger than age two years with HT1 who are treated with nitisinone and a low-tyrosine diet have markedly better outcomes compared to the children treated with a low-tyrosine diet alone [5]. The HT1 patients in the Philippines were all treated within the first three months of life. The majority of the findings consistent with HT1 were limited to laboratory abnormalities alone. Liver and gastrointestinal, kidney, and nervous system manifestations were noted in between 6.25 and 12.5% of the patients.

Based on the onset of symptoms, HT1 may be classified into acute, subacute, and chronic forms. The clinical spectrum varies from acute forms presenting before six months old, subacute forms presenting symptoms between 6 and 12 months old, and chronic forms after 12 months of age [9]. Patients in this cohort who had clinical signs and symptoms (hepatic/gastrointestinal and kidney) presented within the first 6 months of life. Generally, there are no clinical and genotype correlations. The same genotype was observed in unrelated individuals. Acute and chronic forms have been reported in the same families [10].

Among the 16 patients identified with HT1, 14 are alive and asymptomatic. Each of these patients is on regular follow-up with one of the Newborn Screening Continuity Clinics across the country. In the Philippine NBS program, confirmatory testing for HT1 is limited to urine organic acid and plasma amino acid assays. Molecular confirmation is not mandatory. Of the 16 patients, 2 biochemically confirmed patients had an untimely demise due to infection. One died at the age of 1 year 9 months due to severe pneumonia and seizures, while the other died at 1 year 11 months due to sepsis. Although infection remains to be the top cause of mortality in children under five years old, including pneumonia and sepsis [10], the metabolic condition could have contributed to the outcome of these two patients.

As a consequence of early dietary management and NTBC therapy, survival rates of HT1 have improved. Other complications like neurodevelopmental impairments and ophthalmologic concerns are being monitored and have not been reported in this cohort.

In a study on the compilation of all HT1 alleles published worldwide in 2015, three of the pathogenic variants in this cohort were not reported. The other two pathogenic variants in this cohort, c.709C>T and c.82-1G>A, were already reported in previous publications [11]. The FAH c.709C>T (p.Arg237X) is reported to be a common pathogenic variant in patients from the Middle East [12,13], while c.82-1G>A is a splicing pathogenic variant previously reported in Spain [10]. The most common pathogenic variant in our cohort is c.122T>C (p.Leu41Pro). This sequence change replaces leucine, a neutral and nonpolar amino acid, with proline, also a neutral and nonpolar amino acid, at codon 41 of the FAH protein. This variant is not present in population databases. This missense change has been observed in individual(s) with tyrosinemia type 1 via Invitae, where our patients had their molecular confirmation.

The period prevalence of tyrosinemia type 1 in the Philippine NBS program from December 2014 to September 2022 was 1:338,269. This is lower compared to the incidence rate of tyrosinemia I worldwide, which is noted to be approximately 1 in 100,000 births [12]. The Province of Quebec, Canada, recorded a higher-than-expected frequency of tyrosinemia type 1, namely, 1:19,819. The higher-than-average frequency in this area is attributed to the genetic founder effect [14].

This study described the clinical, biochemical, and molecular characteristics of Filipino patients diagnosed with HT1. This may be a basis for future outcome studies using uniform monitoring protocol.

## 5. Conclusions

The expansion of the Philippine newborn screening program in 2014 allowed the early diagnosis and treatment of confirmed HT1 patients. Although most of the patients had biochemical abnormalities, the majority of patients did not present with the devastating complications of HT1. Confirmatory testing via biochemical and/or molecular techniques facilitated early initiation of treatment and improved outcomes. The period prevalence of HT1 cases in the Philippines from December 2014 to September 2022 was 1:338,269.

## Figures and Tables

**Table 1:** General characteristics and biochemical profile of tyrosinemia type 1 patients in the Philippines.

	Current Age	Age at Confirmatory Test	Age at Treatment Initiation	NBS Results	Plasma Amino Acid Profile			Urine Organic Acid Profile				
Patient	Months	Days	Days	Succinylacetone (<5 µmol/L)	Tyrosine (µmol/L) Reference Range: 0–30 days: 55–147 31 days-23 mos: 22–108	Phenylalanine (µmol/L) Reference Range: 0–30 days: 38–137 31 days-23 mos: 31–75	Methionine (µmol/L) Reference Range: 0–30 days: 10–60 31 days-23 mos: 9–42	Succinylacetone	4-hydroxyphenylacetate	4-hydroxyphenyllactate	4-hydroxyphenylpyruvate	N-acetyltyrosine
1	54	27	56	13.9	312	83	40	+	+++	+++	+++	+
2	53	7	35	8.9	1305	296	686	+	+++	+++	+++	+
3	d. 22	7	21	16.4	511	215	429	+	+++	+++	+++	+
4	43	30	36	6.3	371	58	22	- a	+	+	+	+
5	d. 21	8	16	14.1	150	46	13	+	+	+++	+++	-
6	47	8	24	18.8	110	60	26	+	+	+++	+++	+
7	43	13	19	10.8	BAR	BAR	31	+	+	+++	+++	+
8	38	72	80	5.8	784	171	375	++	++	+++	+++	+
9	31	6	8	16.7	217	82	28	+	++	++	++	+
10	31	10	16	10	341	11	20	+	++	+++	+++	+
11	31	31	46	5.4	502	63	37	++	++	+++	+++	+
12	28	6	13	12	1023	265	167	+	+++	+++	+++	+
13	23	4	6	10.2	629	96	32	+	++	+++	+++	+
14	23	22	30	5.2	487	83	40	++	+++	+++	+++	+
15	22	7	11	10.7	292	49	17	+	++	+++	+++	+
16	18	10	11	12	557	94	43	++	+++	+++	+++	+
Mean (SD)	NA	NA	26.8 (±19.4)	11.1 (±4.1)	506.1 (±15.9)	111.5 (±82.1)	125.4 (±190.9)	NA	NA	NA	NA	NA

d.: deceased; BAR: below assay range; a: management already initiated upon testing; SD: standard deviation; +: slightly increased; ++: moderately increased; +++: grossly increased; -: absent; NA: not applicable.

**Table 2:** Clinical characteristics of patients with tyrosinemia type 1 in the Philippines around the time of treatment initiation.

Clinical Characteristic	Number of Patients (*n* = 16)	Percentage (%)
Abnormal clotting times	10	62.5
High alpha fetoprotein	6	37.5
Anemia	5	31.3
Metabolic acidosis	5	31.3
Conjugated hyperbilirubinemia	4	25
Transaminitis	4	25
High alkaline phosphatase	4	25
Jaundice	2	12.5
Liver failure	2	12.5
Rickets	2	12.5
Cirrhosis	1	6.3
Ascites	1	6.3
Tubulopathy	1	6.3
Seizure	1	6.3

**Table 3:** Genotypic frequencies of FAH pathogenic variants in Filipino patients with tyrosinemia type 1 in the study.

FAH Pathogenic Variants	Number of Patients (*n* = 14)	Percentage (%)
Homozygous c.122T>C (p.Leu41Pro)	9	64.3
Homozygous c.535_536del (p.Gln179Aspfs*4)	1	7.1
Homozygous c.235A>T (p.Lys79*)	1	7.1
Homozygous c.709C>T (p.Arg237*)	1	7.1
Compound heterozygous c.122T>C (p.Leu41Pro);c.82-1G>A (p.?)	1	7.1
Compound heterozygous c.122T>C (p.Leu41Pro); c.535_536del (p.Gln179Aspfs*4)	1	7.1

**Table 4:** Allelic frequencies of FAH pathogenic variants in Filipino patients with tyrosinemia type 1 in the study.

FAH Pathogenic Variant	Number of Alleles (*n* = 28)	Percentage (%)
c.122T>C (p.Leu41Pro)	20	71.4
c.535_536del (p.Gln179Aspfs*4)	3	10.7
c.235A>T (p.Lys79*)	2	7.1
c.709C>T (p.Arg237*	2	7.1
c.82-1G>Ap.?	1	3.6

## Data Availability

The original contributions presented in the study are included in the article, further inquiries can be directed to the corresponding author.
